# Activation of non-canonical WNT signaling in human visceral adipose tissue contributes to local and systemic inflammation

**DOI:** 10.1038/s41598-017-17509-5

**Published:** 2017-12-11

**Authors:** María A. Zuriaga, José J. Fuster, Melissa G. Farb, Susan MacLauchlan, Rosa Bretón-Romero, Shakun Karki, Donald T. Hess, Caroline M. Apovian, Naomi M. Hamburg, Noyan Gokce, Kenneth Walsh

**Affiliations:** 1 Molecular Cardiology, Whitaker Cardiovascular Institute, Boston University School of Medicine, 715 Albany Street, W-611 Boston, MA USA; 2 Cardiovascular Medicine, Whitaker Cardiovascular Institute, Boston University School of Medicine, 715 Albany Street, E-7 C.H.U. Boston, MA USA; 3 Department of General Surgery, Boston University School of Medicine, Boston, MA USA; 4 Department of Medicine, Section of Endocrinology, Diabetes and Nutrition, Boston University School of Medicine, Boston, MA USA

## Abstract

The accumulation of visceral adiposity is strongly associated with systemic inflammation and increased cardiometabolic risk. WNT5A, a non-canonical WNT ligand, has been shown to promote adipose tissue inflammation and insulin resistance in animal studies. Among other non-canonical pathways, WNT5A activates planar cell polarity (PCP) signaling. The current study investigated the potential contribution of non-canonical WNT5A/PCP signaling to visceral adipose tissue (VAT) inflammation and associated metabolic dysfunction in individuals with obesity. VAT and subcutaneous adipose tissue (SAT) samples obtained from subjects undergoing bariatric surgery were analyzed by qRT-PCR for expression of WNT/PCP genes. *In vitro* experiments were conducted with preadipocytes isolated from VAT and SAT biopsies. The expression of 23 out of 33 PCP genes was enriched in VAT compared to SAT. Strong positive expression correlations of individual PCP genes were observed in VAT. *WNT5A* expression in VAT, but not in SAT, correlated with indexes of JNK signaling activity, *IL6*, waist-to-hip ratio and hsCRP. *In vitro*, WNT5A promoted the expression of *IL6* in human preadipocytes. In conclusion, elevated non-canonical WNT5A signaling in VAT contributes to the exacerbated IL-6 production in this depot and the low-grade systemic inflammation typically associated with visceral adiposity.

## Introduction

Obesity, defined as a body mass index (BMI) ≥30 kg/m^2^, is a major risk factor for systemic metabolic dysfunction and cardiovascular disease (CVD). However, a wealth of evidence has now confirmed that the regional distribution of adipose tissue is as important, if not more important, than overall adiposity in determining cardiometabolic risk^1^. It is widely accepted that accumulation of intra-abdominal visceral fat is a major contributor to systemic metabolic dysfunction and cardiovascular risk. In this regard, an increasing body of evidence supports the notion that visceral (VAT) and subcutaneous (SAT) adipose tissue exhibit different intrinsic properties, which make VAT a more pathogenic depot^2^. More specifically, the increased cardiometabolic risk associated with visceral adiposity has been linked with an overproduction of pro-inflammatory and pro-atherogenic cytokines, which contribute to establish a low-grade systemic inflammatory state that drives cardiometabolic disease^3–8^. However, the specific signaling pathways in VAT that account for this exacerbated inflammatory response remain largely unexplored.

Emerging evidence suggests that developmental genes play central roles in fat distribution and the different properties of specific adipose tissue depots^9–11^. Wingless-type MMTV integration site family (WNT) proteins are secreted signaling molecules that play fundamental roles during embryonic development and have been implicated in numerous critical aspects of physiology and disease in the adult. There are 19 WNT family members in mammals, which typically act in an autocrine/paracrine fashion and activate a number of different signaling pathways, typically classified as either canonical (β-catenin-dependent) or non-canonical (β-catenin-independent). While the role of canonical WNT signaling in the regulation of adipose tissue expansion is generally accepted^12^, the potential effects of non-canonical WNT signaling activation in this tissue and its contribution to fat depot heterogeneity in humans remain poorly defined. We recently reported that WNT5A, a WNT ligand that predominantly activates non-canonical WNT signaling contributes to obesity-induced adipose tissue inflammation and systemic insulin resistance in obese mice^13,14^. Although WNT5A is able to activate various non-canonical signaling pathways, its roles in embryo development are mostly mediated by activation of the WNT/planar cell polarity (PCP) pathway, which frequently leads to activation of JUN N-terminal kinase (JNK) signaling^15,16^. In adults, WNT/PCP signaling has been shown to contribute to cancer development in different settings, but its specific contribution to other disease processes has just recently begun to be explored^17–22^. The present study provides evidence supporting that WNT5A/PCP signaling is overactivated in VAT compared to SAT and contributes to the increased local and systemic inflammation associated with visceral adiposity.

## Results

### Increased expression of WNT/PCP genes in visceral adipose tissue

**Figure 1:**
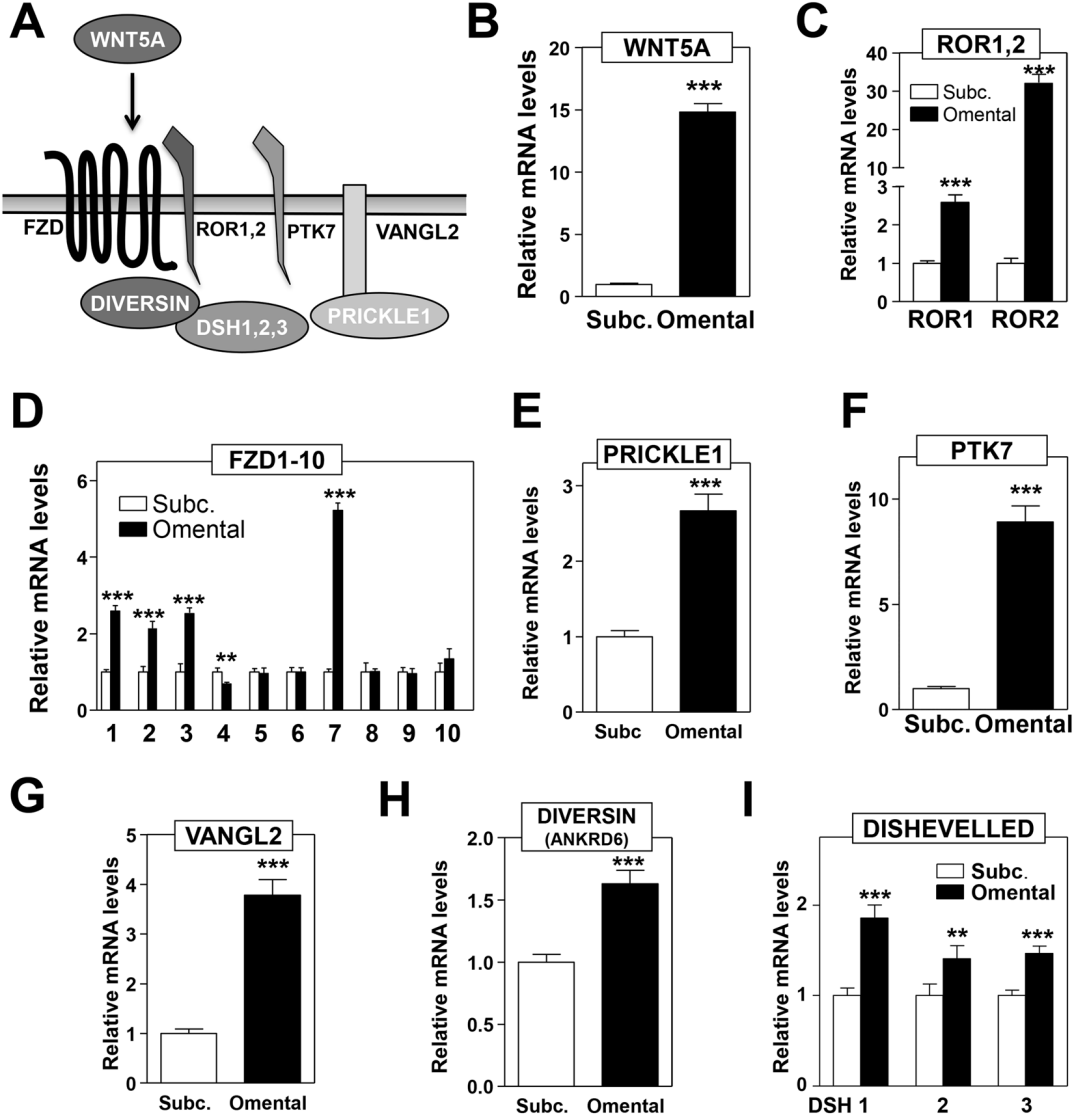
Overexpression of core PCP genes in visceral adipose tissue. (**A**) Schematic representation of main PCP signaling components. (**B–I**) Transcript levels of *WNT5A* (**B**), *ROR* co-receptors (**C**), *FZD* receptors (**D**) and main transmembrane and intracellular PCP signaling mediators (**E**–**I**) were evaluated in subcutaneous and visceral adipose tissue of subjects undergoing bariatric surgery by qRT-PCR analysis (**p < 0.001, ***p < 0.0001).

To investigate the role of WNT5A and downstream signaling in obesity-associated adipose tissue dysfunction in humans, we used quantitative real-time PCR (qRT-PCR) to evaluate transcript expression of 33 genes involved in WNT/PCP signaling in visceral (omental) and subcutaneous fat obtained from 44 individuals at the time of bariatric surgery. The clinical characteristics of this human population are summarized in Supplementary Table S1. This analysis revealed that the expression of 70% of the analyzed genes (23 out of 33) was significantly higher in VAT than in SAT (Table 1). Figure 1 shows the expression of a subset of these genes that represent the main components of the WNT/PCP ligand/receptor complex (Fig. 1A). Consistent with previous reports^14,23^, there was a marked upregulation (14.72-fold) of *WNT5A* expression in VAT compared with SAT (Fig. 1B). The differential expression of *WNT5A* between depots was not affected by age (data not shown), but it was greater in men (VAT vs. SAT fold-change: 14.13 ± 0.76 in women, 20.36 ± 1.6 in men, p = 0.0004) and in subjects with diabetes (VAT vs SAT fold-change: 13.36 ± 0.73 without diabetes, 17.62 ± 1.41 with diabetes, p = 0.0132).

**Table 1:** Relative gene expression of WNT/PCP genes in VAT compared to SAT.

	Gene	Relative gene expression (visceral vs subcutaneous)	p value
WNT ligands	WNT4	4.41 ± 0.73	1.8 × 10^−5^
	WNT5A	14.72 ± 0.66	3.2 × 10^−25^
	WNT9B	1.62 ± 0.24	0.0362
	WNT11	0.94 ± 0.07	0.3974
Transmembrane proteins (receptors, co-receptors)	ROR1	2.52 ± 0.19	2.7 × 10^−11^
	ROR2	33.39 ± 2.1	2.4 × 10^−19^
	FZD1	2.50 ± 0.14	1.2 × 10^−14^
	FZD2	2.13 ± 0.17	2.7 × 10^−8^
	FZD3	2.44 ± 0.14	1.1 × 10^−6^
	FZD4	0.69 ± 0.05	0.0025
	FZD5	0.97 ± 0.07	0.6621
	FZD6	1.00 ± 0.06	0.2090
	FZD7	5.24 ± 0.31	1.4 × 10^−18^
	FZD8	1.02 ± 0.06	0.9688
	FZD9	0.89 ± 0.12	0.2588
	FZD10	1.26 ± 0.24	0.1938
	VANGL1	1.09 ± 0.07	0.5189
	VANGL2	3.85 ± 0.31	1.2 × 10^−13^
	CELSR1	3.37 ± 0.21	2.8 × 10^−15^
	CELSR2	3.31 ± 0.40	5.9 × 10^−10^
	CELSR3	1.6 ± 0.20	6.7 × 10^−5^
	PTK7	9.06 ± 0.89	9.1 × 10^−12^
	RYK	1.18 ± 0.06	0.0260
Intracellular signaling mediators	DVL1	1.74 ± 0.12	1.65 × 10^−11^
	DVL2	1.36 ± 0.13	0.0016
	DVL3	1.45 ± 0.08	9.8 × 10^−7^
	ANKRD6	1.68 ± 0.12	1.9 × 10^−8^
	INVS	1.61 ± 0.08	5.83 × 10^−10^
	SCRIB	2.02 ± 0.07	1.87 × 10^−17^
	PRICKLE1	2.60 ± 0.20	1.34 × 10^−11^
	PRICKLE2	1.07 ± 0.05	0.3859
	DAAM1	1.15 ± 0.07	0.0740
	DAAM2	1.04 ± 0.07	0.6014

Transcript levels were evaluated by qRT-PCR. Data is expressed as Mean ± SEM.

Many signaling genes immediately downstream of *WNT5A* in the PCP pathway were markedly and uniformly overrepresented in VAT. FRIZZLED (FZD) proteins are the main receptors for WNT ligands, and many *FZD* genes, including *FZD1*, 2, 3 and 7, were overrepresented 2- or more-fold in VAT compared with SAT (Fig. 1D). Furthermore, there was a 33-fold overrepresentation of receptor tyrosine kinase-like orphan receptor 2 (*ROR2*) (Fig. 1C), one of the co-receptors for WNT5A-induced non-canonical WNT/PCP signaling^24–26^. The transcript expression of PTK7, a membrane protein that contributes to WNT5A/ROR2 signaling in mammalian cells^27^, was also markedly overrepresented in VAT (Fig. 1F). Similarly, we found a more modest, but clearly statistically significant elevation of the expression of other main components of the WNT/PCP core receptor complex in VAT including *VANGL2*, Diversin (*ANKRD6*), Dishevelled (*DSH*) 1, 2 and 3, and *PRICKLE1* (Fig. 1E,G,H,I and Table 1).

With the exception of *WNT5A*, the increased expression of PCP genes in VAT was not affected by gender or diabetes status (data not shown). However, there were strong positive correlations between the expressions of individual WNT/PCP receptor components in VAT. Expression levels of *ROR2*, *FZD7*, *VANGL2* and *PRICKLE1* correlated with each other (Supplementary Fig. S1), and these correlations were statistically significant in subjects with and without diabetes, although in most cases they were much stronger in the group with diabetes (Supplementary Table S2). Expression of PCP receptor components in VAT also correlated with that of key intracellular PCP signaling modulators, although almost exclusively in the subjects with diabetes. Specifically, VAT expression of *ROR2*, *FZD7*, *VANGL2* and *PRICKLE1* correlated with that of *DSH1*,*2*,*3* and *DIVERSIN/ANKRD6* in the diabetes group, but not in most cases in the group without diabetes (Supplementary Table S2). In addition, *WNT5A* expression levels in VAT correlated with those of some of the PCP signaling components, such as *VANGL2*, *PRICKLE1*, *DSH1*,*2*,*3* and *DIVERSIN/ANKRD6* only in subjects with diabetes (Supplementary Table S2). Some of the correlations among PCP genes were also observed in SAT, although these were overall less frequent and less statistically significant than in VAT, and were not consistently affected by diabetes (data not shown). Collectively, these data suggest the existence of common regulatory mechanisms that allow the coordinated upregulation of WNT/PCP genes in visceral fat depots, particularly under diabetic conditions.

### Increased *WNT5A* expression in visceral fat is associated with augmented JNK signaling

**Figure 2:**
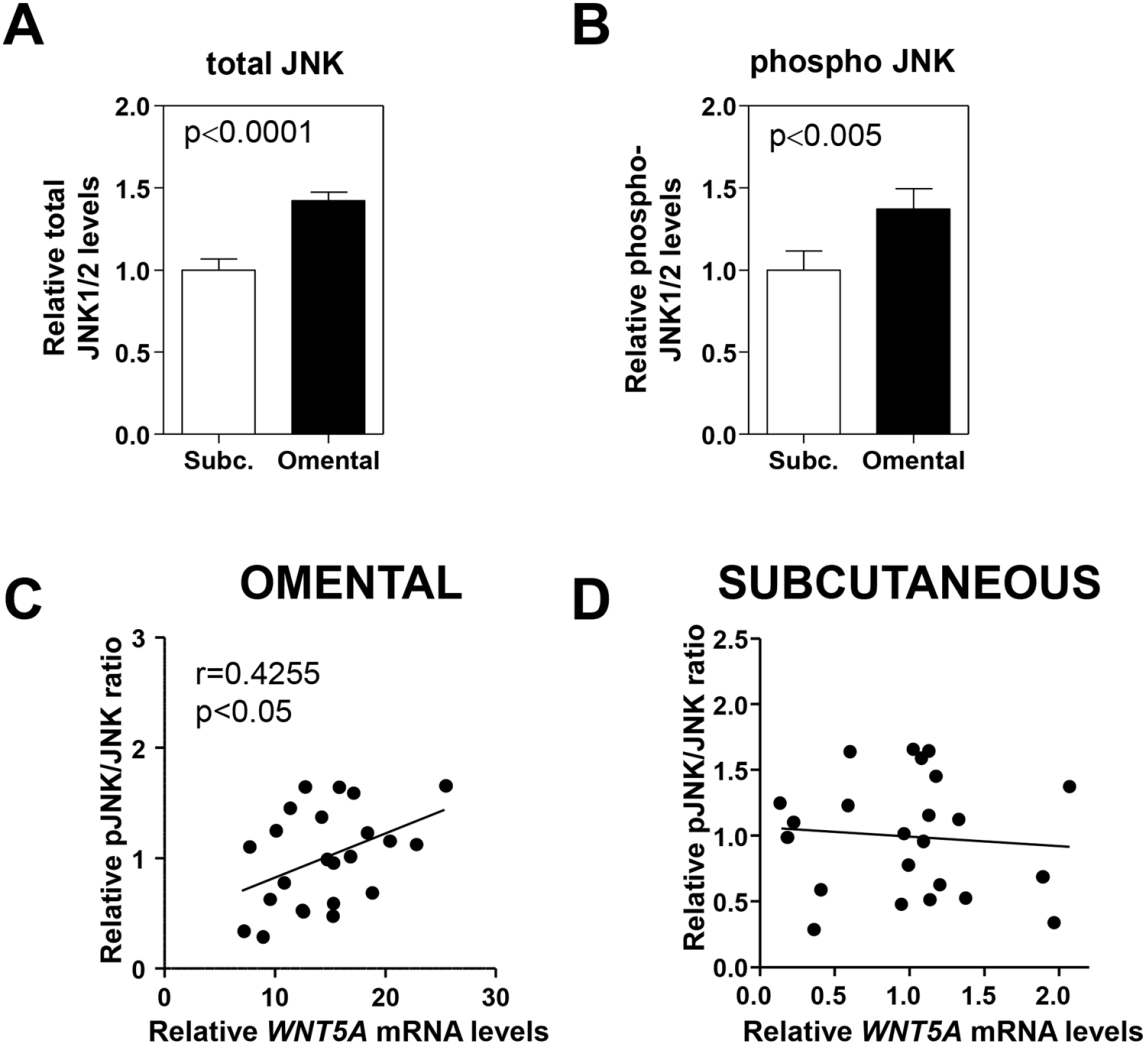
*WNT5A* expression correlates with JNK signaling activity in VAT. (**A**,**B**) Relative levels of total (**A**) and phosphorylated JNK1/2 (**B**) in SAT and VAT were quantified by ELISA. (**C**,**D**) Pearson’s coefficients (r) were used to analyze the correlation between *WNT5A* transcript levels and pJNK/JNK protein ratios in visceral/omental (**C**) and subcutaneous (**D**) fat.

WNT/PCP signaling frequently leads to the activation of JNK signaling^15,16^, a major driver of adipose tissue dysfunction and associated systemic metabolic abnormalities in animal models of obesity^28,29^. Therefore, the extent of JNK signaling was evaluated in VAT and SAT. JNK1/2 protein expression and phosphorylation were quantified by an enzyme-linked immunosorbent assay in a subset of fat samples for which protein extracts were available (n = 23). Consistent with a previous report^30^, we found increased levels of both phosphorylated and total JNK1/2 in VAT (Fig. 2A,B), suggesting increased JNK signaling activity in this depot. Notably, a statistically significant correlation was found between *WNT5A* transcript expression and the phosphorylated JNK/total JNK ratio, a widely used index of JNK signaling activity (Fig. 2C). Such a correlation was not observed in SAT (Fig. 2D). These data suggest that WNT5A is a regulator of JNK signaling in adipose tissue, and that increased WNT5A levels in VAT contribute to the increased JNK signaling activity observed in this depot.

### Correlation between *WNT5A* and Interleukin-6 expression in visceral fat

**Figure 3:**
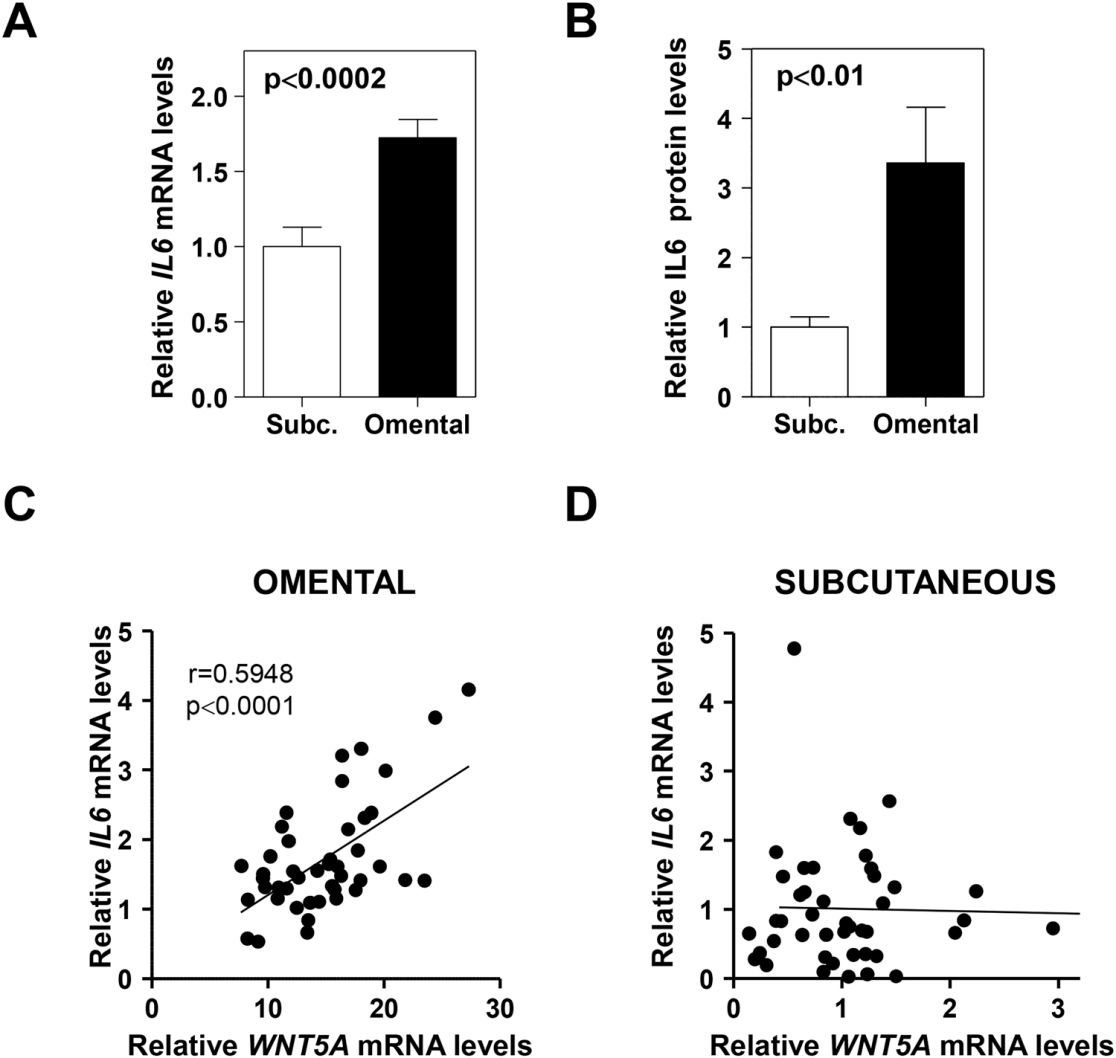
*WNT5A* expression correlates with *IL6* expression in VAT. (**A**,**B**) *IL6* transcript and protein levels in SAT and VAT were evaluated by qRT-PCR (**A**) or ELISA (**B**). (**C**,**D**) Pearson’s correlation coefficients (r) were used to analyze the correlation between *WNT5A* and *IL6* transcript levels in omental (**C**) and subcutaneous (**D**) fat were evaluated by qRT-PCR.

JNK signaling in adipose tissue has been linked to interleukin 6 (IL-6) expression in experimental models^29^, and our previous mouse studies suggested a main role for exacerbated IL-6 production in mediating the pathogenic actions of WNT5A in the setting of obesity-induced adipose tissue dysfunction and associated metabolic abnormalities^14^. Furthermore, IL-6 has been suggested to be an important driver of metabolic dysfunction associated with visceral adiposity in humans^4,8,31^. Based on these considerations, we next evaluated whether increased *WNT5A* expression in human VAT is associated with *IL6* expression in this depot. *IL6* expression was significantly increased in VAT compared to SAT both at the transcript (Fig. 3A) and protein (Fig. 3B) level. Notably, a highly statistically significant correlation between the transcript expression of *WNT5A* and *IL6* was observed in VAT (Fig. 3C), but not in SAT (Fig. 3D). Furthermore, this correlation was substantially tighter in subjects with diabetics (Supplementary Table S3). *IL6* transcript levels in VAT also correlated with the expression of the intracellular PCP signaling modulators *DHS1*,*2*,*3* in subjects with diabetes, but not in those without diabetes (Supplementary Table S3). No correlations were observed in SAT regardless of diabetes status (Fig. 3D and data not shown). Overall, these data suggest that increased *WNT5A* signaling in VAT specifically contributes to the elevated production of IL-6 in this depot, which has been previously linked with the systemic metabolic alterations associated with visceral adiposity^4^.

### WNT5A promotes IL6 mRNA expression in human preadipocytes

**Figure 4:**
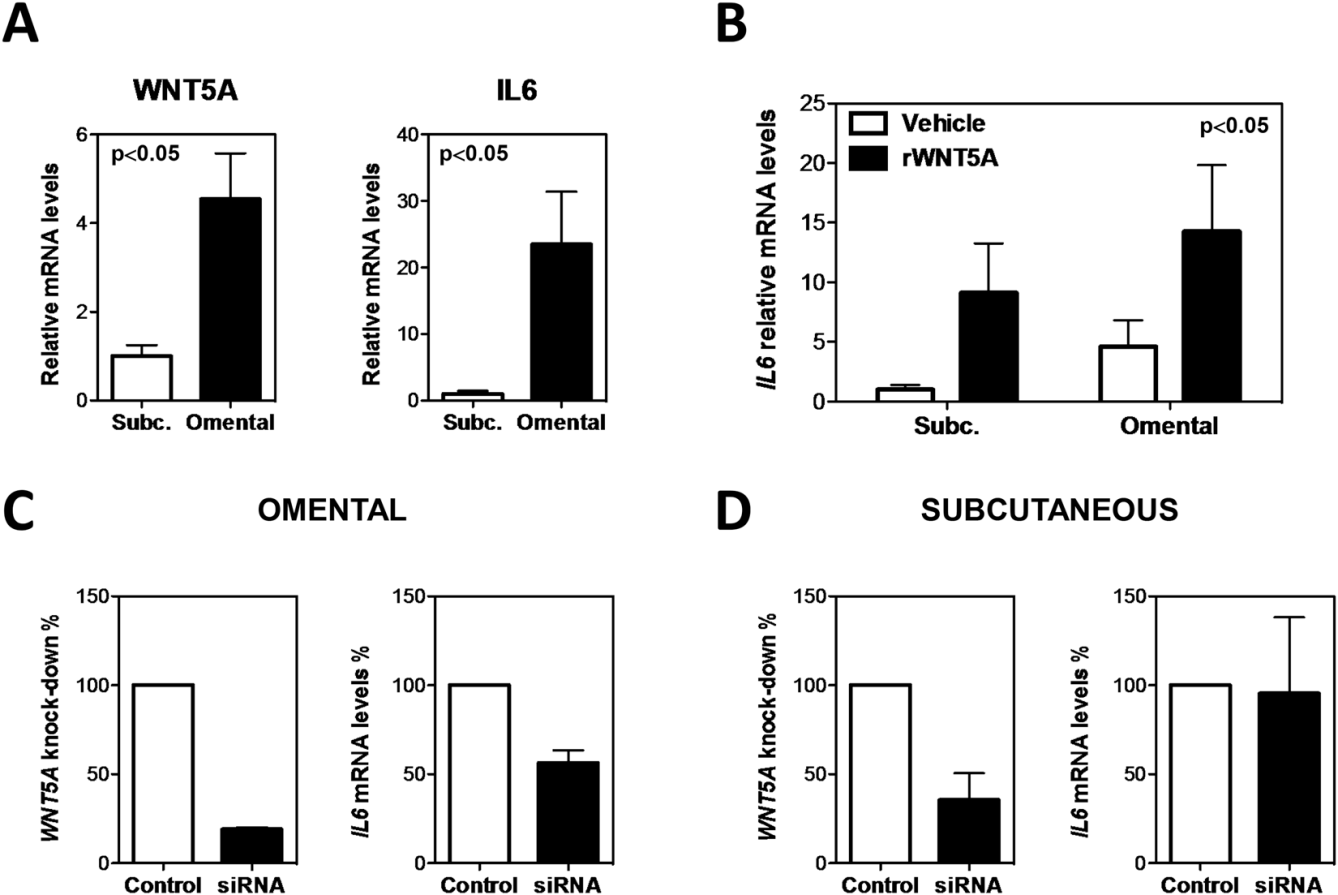
*WNT5A* promotes *IL6* production in human preadipocytes *in vitro*. (**A**) Baseline mRNA expression of *WNT5A* and *IL6* was measured in visceral/omental and subcutaneous preadipocytes by qRT-PCR (*n* = 5). (**B**) Transcript levels of *IL6* after treatment with recombinant WNT5A protein (1 µg/ml for 8 hours), assessed by qRT-PCR analysis. (**C**,**D**) Transcript levels of *IL6* after treatment with with siRNA against *WNT5A* (**C**) or scrambled siRNA (**D**) as control. Data represented as percentage of *WNT5A* knock-down and percentage of change in *IL6* mRNA levels.

To investigate if WNT5A is causally linked to IL-6 expression, we evaluated whether treatment with exogenous WNT5A or *WNT5A* knockdown affect *IL6* transcript levels in human primary preadipocytes. Supporting the validity of this cell culture model, we observed that transcript levels of both *WNT5A* and *IL6* are higher in omental versus subcutaneous preadipocytes, consistent with their higher expression in VAT (Fig. 4A). Treatment with human recombinant WNT5A protein increased *IL6* transcript levels in both subcutaneous and omental cells, and brought *IL6* levels in subcutaneous cells to approximately the same level observed in omental cells at baseline conditions (Fig. 4B). Treatment with siRNA against *WNT5A* mRNA diminished *WNT5A* transcript levels by 81% in omental cells, and this led to a 43% decrease of *IL6* mRNA levels, supporting a direct effect of WNT5A on IL-6 expression (Fig. 4C). *WNT5A* knock-down was more modest in subcutaneous cells (64%) and this did not have a statistically significant effect on *IL6* transcript levels (Fig. 4D).

### WNT5A expression in visceral fat is associated with markers of systemic inflammation and cardiometabolic risk

**Figure 5:**
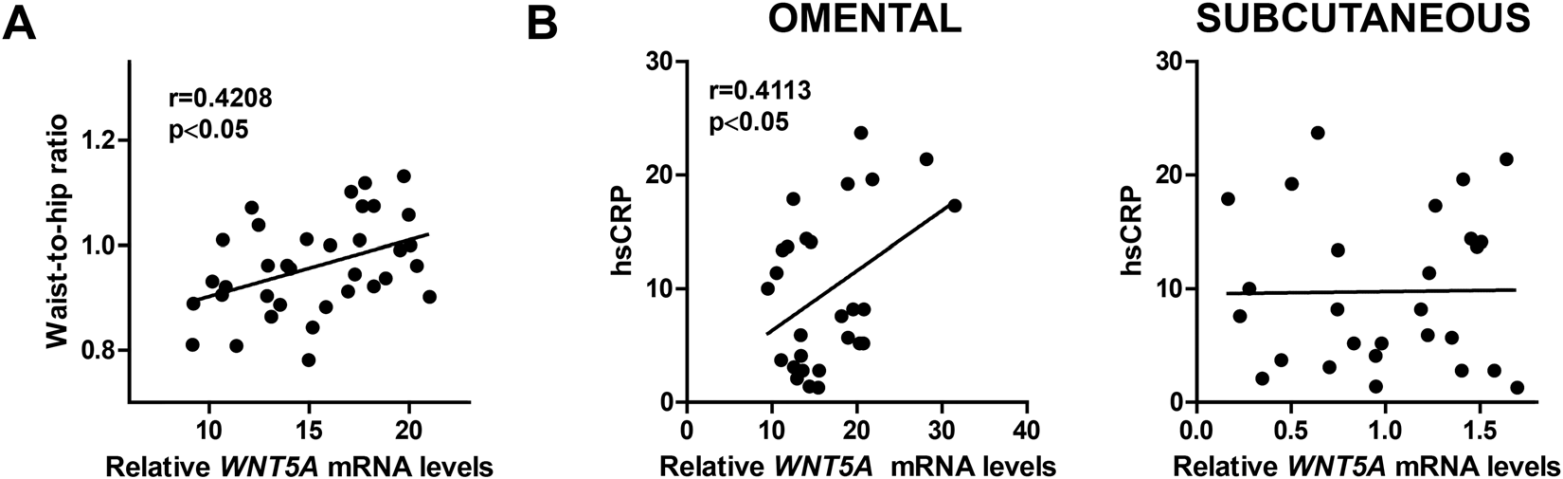
Positive correlation between *WNT5A* expression in VAT and markers of systemic inflammation and cardiometabolic risk under obesity conditions. Pearson’s coefficients (r) were used to analyze the correlation between *WNT5A* transcript levels and waist-to-hip ratio (**A**) and hsCRP (**B**).

Abdominal adiposity is associated with a low-grade systemic inflammatory response, which has been linked to increased IL-6 production in visceral fat depots^4,31,32^. Therefore, we next investigated whether the expression of *WNT5A* correlated with markers of systemic inflammation. In agreement with previous studies by our group and others^14,23^, we observed a correlation between *WNT5A* expression in VAT and waist-to-hip ratio, an anthropometric indicator of abdominal obesity (Fig. 5A). This correlation remained statistically significant in subjects with diabetes, but not in subjects without it (Supplementary Table S3). *WNT5A* was the only PCP gene whose expression in VAT correlated with waist-to-hip ratio (Supplementary Table S3). A statistically significant correlation between *WNT5A* expression in VAT and circulating C-reactive protein (CRP) levels was observed after exclusion of patients on anti-inflammatory drugs (NSAIDs and immunosuppressive drugs) (Fig. 5B). This correlation lost its statistical significance in the group with diabetes, likely due to the reduction in sample size associated with stratification, although a borderline significant trend was observed (r = 0.54, p = 0.056; Supplementary Table S3). In contrast, no correlation or trend were observed in the group without diabetes (r = 0.008, p = 0.97; Supplementary Table S3). Similar trends or correlations with CRP levels where observed when analyzing the expression of *DSH1*,*2*,*3* in VAT (Supplementary Table S3). No correlations with CRP levels were detected in SAT, regardless of diabetes status (Fig. 5B and data not shown). IL-6 expression in VAT and SAT correlated with circulating CRP levels (Supplementary Fig. S2), consistent with the known role of IL-6 as a direct inducer of the expression of CRP in the liver. Overall, these data suggest that increased WNT5A expression in visceral fat depots contributes to the low-grade systemic inflammation typically associated with obesity and visceral adiposity.

## Discussion

The differential expression of developmental genes has been suggested to contribute to the qualitative differences between visceral and subcutaneous adipose tissue and to the different cardiometabolic risk associated with the expansion of these depots in the context of obesity^9–11^. In this study we analyzed gene expression patterns in visceral and subcutaneous adipose tissue to gain insight into the potential role of non-canonical WNT5A/PCP signaling in adipose tissue inflammation in the setting of obesity. Our data show an over-activation of this signaling pathway in VAT under obesity conditions, which may contribute to the exacerbated local and systemic inflammation associated with visceral adiposity and its metabolic and cardiovascular complications.

We and others have previously reported that WNT5A is expressed at higher levels in VAT than SAT in the setting of obesity^14,23^, a finding that we corroborated in the current study. However, the expression of the many signaling genes that modulate WNT5A-mediated PCP signaling had remained largely unexplored. In this regard, our study adds insight into the role of non-canonical WNT signaling in adipose tissue heterogeneity by demonstrating that most of the downstream components of the non-canonical WNT/PCP signaling pathway (70% of the analyzed genes) are markedly overrepresented in VAT compared to SAT. These data suggest that the over-activation of this signaling pathway extends beyond the increased expression of the WNT5A ligand in this depot. It is noteworthy that many of the WNT/PCP receptor components that we found most highly upregulated in VAT (*ROR2* ~33-fold; *PTK7*, ~9-fold; *VANGL2*, ~4-fold; *PRICKLE1*, ~3-fold) have been shown to be essential for WNT5A signaling in different experimental settings^24,27,33^. We are unaware of any other signaling pathway that has been reported to be so uniformly and robustly upregulated in visceral fat. These findings suggest that PCP signaling modulators represent unique nodal drivers of VAT dysfunction in the setting of obesity.

Our data also reveals a remarkable correlation among the expression of different PCP genes in VAT, particularly in subjects with diabetes, suggesting the existence of common regulatory mechanisms that coordinate their expression. The regulators of PCP gene expression in omental fat are probably multiple. Omental and subcutaneous fat have different embryological origins, and this could contribute to the differential expression of WNT/PCP genes. In this regard, the greater expression of WNT5A observed in omental versus subcutaneous preadipocytes is consistent with this possibility. Several developmental genes have been reported to be differentially expressed in the different adipose tissue depots^9–11,34^, although not to the extent that is observed for some WNT/PCP genes. On the other hand, we have previously reported that myeloid cells are a significant source of Wnt5a in mouse adipose tissue^14^, and pro-inflammatory TLR signaling has been reported to increase WNT5A expression in human monocyte/macrophages^35^. Thus, the differential expression of WNT5A in VAT and SAT could also reflect differences in macrophage infiltration and/or activation. Regardless of the underlying mechanism, these data suggest that not only VAT does produce more WNT5A, but also that this fat depot is likely to be more responsive to its actions, given its higher expression of WNT/PCP receptors and other WNT signaling intermediates. Consistent with this notion, we find that WNT5A is associated with increased JNK signaling in VAT, but not in SAT. Although WNT5A is able to activate a plethora of signaling pathways in a context-dependent manner, JNK is the main signaling kinase activated by the WNT/PCP pathway in mammals^15,16^, and it frequently mediates the effects of WNT5A. Notably, experimental studies suggest that JNK signaling is particularly relevant in the setting of obesity-induced inflammation and associated metabolic dysfunction^28,29^.

Systemic low-grade inflammation is one of the main mechanisms that link obesity with insulin resistance, diabetes and cardiovascular disease. While conflicting data have been reported on the contribution of subcutaneous fat, a consistent body of evidence suggests a strong association between the accumulation of intra-abdominal visceral fat and systemic inflammation^36^. The pro-inflammatory cytokine IL-6 is generally believed to be a particularly relevant contributor to the high cardiometabolic risk associated with intra-abdominal fat accumulation. It is produced at higher levels in VAT compared to SAT^3,6,7^ and circulating IL-6 levels exhibit a stronger association with visceral adiposity than other pro-inflammatory cytokines, such as TNF^4,31^. Despite of these findings, the specific mechanisms that account for the increased production of IL-6 in VAT remain poorly defined. In this regard, we recently reported that Wnt5a promotes adipose tissue inflammation and systemic insulin resistance in mouse models of obesity, at least in part through an increased production of IL-6 in adipose tissue^14^. However, the extrapolation of these mouse results to human obesity remained challenging due to the many differences between adipose tissue depots in mice and humans^37^. While omental fat is the prototypical visceral fat depot in humans, this depot is essentially absent in mice. Furthermore, inflammatory responses in mice and humans are known to differ significantly^38^, and the biology of IL-6 is likely to exhibit many differences between species, as suggested by the low amino acid sequence identity between human and mouse IL-6 proteins (~41%)^39^. The results of the current studies with subcutaneous and omental preadipocytes in culture and the *in vivo* association between the expression of *WNT5A* and *IL6* in VAT presented herein supports our previous mouse studies^14^, and provides evidence that WNT5A/PCP signaling is a clinically-relevant contributor to the elevated IL-6 production in VAT. The correlation observed in VAT between *WNT5A* and the pJNK/JNK ratio, an index of JNK signaling, also supports this possibility. JNK signaling is known to promote IL-6 expression in adipose tissue^29^, and our previous mouse studies suggest that JNK signaling is a main mechanism linking WNT5A to IL-6 production^13,14^. Further supporting this WNT5A/IL-6 connection, we also observed a significant association between WNT5A expression in VAT and circulating levels of CRP, a marker of systemic inflammation. The connection between *WNT5A* expression in VAT and IL-6/CRP levels is of particular relevance, given the large body of clinical evidence that links increased IL-6 signaling and/or increased circulating CRP levels to insulin resistance, diabetes and atherosclerotic CVD^4,40–43^. Some of our data suggest that the contribution of non-canonical WNT5A signaling to IL-6 production and inflammation may be particularly relevant in subjects with diabetes, which typically exhibit high levels of systemic inflammation. However, the differences between subjects with and without diabetes observed in our study need to be interpreted with caution, given our limited sample size and the substantial differences in clinical characteristics between subjects with and without diabetes. Future studies with greater sample size will be needed to assess whether a direct connection between diabetes and WNT5A expression in VAT exists in humans, as suggested by our previous mouse studies^14^.

Our study is limited by its cross-sectional nature and the specific characteristics of the studied patient population, which included mostly patients with BMI ≥ 35 kg/m^2^. The analysis of such population may preclude the detection of pathophysiologically relevant associations that might be clearer in more diverse populations. Another limitation of our study is that most patients were on treatment with a number of drugs, including thiazolidinediones, anti-inflammatory drugs and statins, which may have confounded our results. Indeed, the association between *WNT5A* expression in VAT and circulating CRP levels only reached statistical significance after exclusion of subjects on anti-inflammatory drugs. Similarly, we cannot discard that the low calorie diet and associated body weight loss that is required prior to bariatric surgery may have affected gene expression or systemic inflammation in the subjects included in our study. Overall, these limitations highlight the need for more extensive studies that include the analysis of PCP gene expression in VAT and/or SAT of individuals across different BMI groups and before and after bariatric surgery. Future studies are also warranted to evaluate the potential association between WNT5A/PCP signaling in adipose tissue and atherosclerotic CVD, given the major role that IL-6 signaling plays in this condition as revealed by large Mendelian randomization studies^44,45^.

In summary, the present study reveals a remarkable overactivation of non-canonical WNT5A/PCP signaling in visceral fat under obesity conditions, which contributes to the higher IL-6 production and systemic inflammation typically associated with visceral adiposity, in particular in individuals with diabetes. Future studies are warranted to evaluate the mechanisms underlying PCP signaling activation in visceral adipose tissue and the therapeutic or prognostic potential of this pathway in the setting of obesity and associated cardiometabolic disease.

## Methods

### Human adipose tissue samples

Consecutive men and women with obesity (n = 44, BMI ≥35 kg/m^2^, age >18 years) who were referred to undergo bariatric weight loss surgery at Boston Medical Center were recruited for this study. Subjects with unstable medical conditions such as active coronary syndromes, congestive heart failure, systemic infection, acute illness, malignancy or pregnancy were excluded from participation. Anthropometric measures and blood draws were obtained during a single baseline pre-surgical visit, which occurred within 1–2 weeks before their planned surgery and adipose tissue collection. Clinical characteristics including blood pressure, heart rate, height, weight, BMI, and waist circumference were recorded by established methods. Subjects were in a fasting state and their medications held in the morning when blood samples were drawn for biochemical analyses including glucose and insulin levels at the time of the baseline pre-surgical visit. Subjects were diagnosed with diabetes if they had fasting plasma glucose ≥126 mg/dl and/or HbA1C ≥6.5%, were taking glucose lowering medications or had history of diabetes. Study population characteristics are summarized in Supplementary Table S1. Subcutaneous and visceral adipose tissue biopsies were collected intraoperatively during bariatric surgery from a total of 44 men and women. Subcutaneous adipose tissue was collected from the lower abdominal wall and visceral tissue from the greater omentum. The study was approved by Boston Medical Center Institutional Review Board and it was conducted in accordance with the Declaration of Helsinki. All subjects gave written informed consent.

### Gene expression analysis

Adipose tissue samples were collected in RNAlater RNA Stabilization Reagent (Qiagen) and stored at −80 °C. mRNA of whole adipose tissue samples was isolated using RNeasy Lipid Tissue Mini Kit (Qiagen) and RNA quality was confirmed by gel electrophoresis. Reverse transcription to cDNA was performed with High Capacity cDNA Reverse Transcription Kit (Applied Biosystems), and a pre-amplification step of selected target genes was conducted using TaqMan PreAmp Master Mix and specific TaqMan gene expression assays (Applied Biosystems). Real-time PCR was performed in a Viia7 thermal cycler (Applied Biosystems) using TaqMan gene expression assays and TaqMan Gene Expression Master Mix. Expression data for all target genes was normalized to *GAPDH* expression, analyzed using the ΔΔCt method and expressed as fold change of visceral adipose tissue versus subcutaneous adipose tissue. Samples were not pooled for this analysis and expression of all target genes was measured in both VAT and SAT samples for all the subjects included in the study.

### Protein analysis

Protein extracts from human fat samples were obtained by tissue homogenization in a TissueLyser II disruptor (Qiagen) in the presence of ice-cold lysis buffer (Cell Signaling Technologies) supplemented with protease/phosphatase inhibitors (Roche Applied Science). Commercially available ELISA kits were used to quantify relative levels of phosphorylated and total JNK1/2 (Cell Signaling Technologies) and IL-6 (R&D Systems) following manufacturer’s instructions.

### Cell culture

Preadipocytes isolated from subcutaneous and omental adipose tissue biopsies obtained from individuals with obesity during bariatric surgery were purchased from the Boston Nutrition and Obesity Research Center (BNORC). Cells isolated from 5 patients were used for the *in vitro* experiments. Cells on passage 4 were treated with recombinant human recombinant WNT5A protein (R&D) or vehicle as control, and siRNA against WNT5A or scrambled siRNA as control (GE Dharmacon, ON-TARGETplus siRNA and DharmaFECT 1 Transfection Reagent). RNA from cells was isolated using RNeasy Micro Kit (Qiagen). cDNA synthesis and RT-PCR to asses *WNT5A* and *IL6* mRNA levels were performed as described above (without pre-amplification).

### Statistical analysis

Data are shown as mean ± SEM unless otherwise stated. Statistical significance of differences between gene expression in omental and subcutaneous fat were assessed by paired Student’s *t* tests. Pearson’s correlation coefficients (r) were used to analyze the association between variables. A two-way ANOVA was used for the rWNT5A *in vitro* study. All statistical tests were performed using GraphPad Prism software (GraphPad Software Inc.).

### Data availability statement

The datasets generated during and/or analysed during the current study are available from the corresponding author on reasonable request.

## Electronic supplementary material

Supplementary Information

## References

[1] Després J-P (2012). Body Fat Distribution and Risk of Cardiovascular Disease: An Update. Circulation.

[2] Tchkonia T (2013). Mechanisms and Metabolic Implications of Regional Differences among Fat Depots. Cell Metabolism.

[3] Fain JN, Madan AK, Hiler ML, Cheema P, Bahouth SW (2004). Comparison of the release of adipokines by adipose tissue, adipose tissue matrix, and adipocytes from visceral and subcutaneous abdominal adipose tissues of obese humans. Endocrinology.

[4] Cartier A (2008). Visceral obesity and plasma glucose-insulin homeostasis: contributions of interleukin-6 and tumor necrosis factor-alpha in men. J Clin Endocrinol Metab.

[5] Farb MG (2012). Arteriolar function in visceral adipose tissue is impaired in human obesity. Arterioscler Thromb Vasc Biol.

[6] Fontana L, Eagon JC, Trujillo ME, Scherer PE, Klein S (2007). Visceral fat adipokine secretion is associated with systemic inflammation in obese humans. Diabetes.

[7] Fried SK, Bunkin DA, Greenberg AS (1998). Omental and subcutaneous adipose tissues of obese subjects release interleukin-6: depot difference and regulation by glucocorticoid. J Clin Endocrinol Metab.

[8] Fuster JJ, Ouchi N, Gokce N, Walsh K (2016). Obesity-Induced Changes in Adipose Tissue Microenvironment and Their Impact on Cardiovascular Disease. Circ Res.

[9] Gesta S (2006). Evidence for a role of developmental genes in the origin of obesity and body fat distribution. Proc Natl Acad Sci USA.

[10] Yamamoto Y (2010). Adipose depots possess unique developmental gene signatures. Obesity (Silver Spring).

[11] Brune, J. E. *et al*. Fat depot-specific expression of HOXC9 and HOXC10 may contribute to adverse fat distribution and related metabolic traits. *Obesity* (*Silver Spring*), 10.1002/oby.21317 (2015). 10.1002/oby.21317 26647900

[12] Christodoulides C, Lagathu C, Sethi JK, Vidal-Puig A (2009). Adipogenesis and WNT signalling. Trends Endocrinol Metab.

[13] Ouchi N (2010). Sfrp5 is an anti-inflammatory adipokine that modulates metabolic dysfunction in obesity. Science.

[14] Fuster JJ (2015). Noncanonical wnt signaling promotes obesity-induced adipose tissue inflammation and metabolic dysfunction independent of adipose tissue expansion. Diabetes.

[15] Boutros M, Paricio N, Strutt DI, Mlodzik M (1998). Dishevelled activates JNK and discriminates between JNK pathways in planar polarity and wingless signaling. Cell.

[16] Gros J (2010). WNT5A/JNK and FGF/MAPK pathways regulate the cellular events shaping the vertebrate limb bud. Curr Biol.

[17] Breton-Romero R (2016). Endothelial Dysfunction in Human Diabetes Is Mediated by Wnt5a-JNK Signaling. Arterioscler Thromb Vasc Biol.

[18] Farb MG (2016). WNT5A-JNK regulation of vascular insulin resistance in human obesity. Vasc Med.

[19] Karki S (2017). WNT5A regulates adipose tissue angiogenesis via antiangiogenic VEGF-A165b in obese humans. Am J Physiol Heart Circ Physiol.

[20] Kikuchi R (2014). An antiangiogenic isoform of VEGF-A contributes to impaired vascularization in peripheral artery disease. Nat Med.

[21] MacLauchlan S (2017). Genetic deficiency of Wnt5a diminishes disease severity in a murine model of rheumatoid arthritis. Arthritis Res Ther.

[22] Nakamura K (2016). Secreted Frizzled-related Protein 5 Diminishes Cardiac Inflammation and Protects the Heart from Ischemia/Reperfusion Injury. J Biol Chem.

[23] Catalan V (2014). Activation of noncanonical Wnt signaling through WNT5A in visceral adipose tissue of obese subjects is related to inflammation. J Clin Endocrinol Metab.

[24] Gao B (2011). Wnt signaling gradients establish planar cell polarity by inducing Vangl2 phosphorylation through Ror2. Dev Cell.

[25] Grumolato L (2010). Canonical and noncanonical Wnts use a common mechanism to activate completely unrelated coreceptors. Genes Dev.

[26] Nomachi A (2008). Receptor tyrosine kinase Ror2 mediates Wnt5a-induced polarized cell migration by activating c-Jun N-terminal kinase via actin-binding protein filamin A. J Biol Chem.

[27] Martinez S (2015). The PTK7 and ROR2 receptors interact in the vertebrate WNT/PCP pathway. J Biol Chem.

[28] Han MS (2013). JNK expression by macrophages promotes obesity-induced insulin resistance and inflammation. Science.

[29] Sabio G (2008). A stress signaling pathway in adipose tissue regulates hepatic insulin resistance. Science.

[30] Bashan N (2007). Mitogen-activated protein kinases, inhibitory-kappaB kinase, and insulin signaling in human omental versus subcutaneous adipose tissue in obesity. Endocrinology.

[31] Park HS, Park JY, Yu R (2005). Relationship of obesity and visceral adiposity with serum concentrations of CRP, TNF-alpha and IL-6. Diabetes Res Clin Pract.

[32] Rexrode KM, Pradhan A, Manson JE, Buring JE, Ridker PM (2003). Relationship of total and abdominal adiposity with CRP and IL-6 in women. Ann Epidemiol.

[33] Liu C (2014). Null and hypomorph Prickle1 alleles in mice phenocopy human Robinow syndrome and disrupt signaling downstream of Wnt5a. Biol Open.

[34] Vohl MC (2004). A survey of genes differentially expressed in subcutaneous and visceral adipose tissue in men. Obes Res.

[35] Blumenthal A (2006). The Wingless homolog WNT5A and its receptor Frizzled-5 regulate inflammatory responses of human mononuclear cells induced by microbial stimulation. Blood.

[36] Despres JP (2012). Abdominal obesity and cardiovascular disease: is inflammation the missing link?. Can J Cardiol.

[37] Zuriaga MA, Fuster JJ, Gokce N, Walsh K (2017). Humans and Mice Display Opposing Patterns of “Browning” Gene Expression in Visceral and Subcutaneous White Adipose Tissue Depots. Front Cardiovasc Med.

[38] Seok J (2013). Genomic responses in mouse models poorly mimic human inflammatory diseases. Proc Natl Acad Sci USA.

[39] Fuster JJ, Walsh K (2014). The good, the bad, and the ugly of interleukin-6 signaling. EMBO J.

[40] Bastard JP (2000). Elevated levels of interleukin 6 are reduced in serum and subcutaneous adipose tissue of obese women after weight loss. J Clin Endocrinol Metab.

[41] Fernandez-Real JM (2001). Circulating interleukin 6 levels, blood pressure, and insulin sensitivity in apparently healthy men and women. J Clin Endocrinol Metab.

[42] Kern PA, Ranganathan S, Li C, Wood L, Ranganathan G (2001). Adipose tissue tumor necrosis factor and interleukin-6 expression in human obesity and insulin resistance. Am J Physiol Endocrinol Metab.

[43] Pradhan AD, Manson JE, Rifai N, Buring JE, Ridker PM (2001). C-reactive protein, interleukin 6, and risk of developing type 2 diabetes mellitus. JAMA.

[44] Collaboration, I. R. G. C. E. R. F. (2012). Interleukin-6 receptor pathways in coronary heart disease: a collaborative meta-analysis of 82 studies. Lancet.

[45] Niu W (2012). Association of interleukin-6 circulating levels with coronary artery disease: a meta-analysis implementing mendelian randomization approach. Int J Cardiol.

