# Supplementary material for: Activation of non-canonical WNT signaling in human visceral adipose tissue contributes to local and systemic inflammation
Source: Sci Rep. 2017 Dec 11;7:17326. doi: 10.1038/s41598-017-17509-5 (PMC5725530; doi:10.1038/s41598-017-17509-5)
Supplement: Supplementary file 1 — Supplementary Information [file 41598_2017_17509_MOESM1_ESM.pdf]

REVISED MANUSCRIPT SREP-17-42474

SUPPLEMENTARY INFORMATION

Activation of non-canonical WNT signaling in human visceral adipose tissue contributes to local and systemic inflammation

María A. Zuriaga[1], José J. Fuster[1,] Melissa G. Farb[2], Susan MacLauchlan[1], Rosa Bretón-Romero[2], Shakun Karki[2], Donald T. Hess[3], Caroline M. Apovian[4], Naomi M. Hamburg[2], Noyan Gokce[2] and Kenneth Walsh[1*]

[1]Molecular Cardiology, Whitaker Cardiovascular Institute, Boston University School of Medicine, 715 Albany Street, W-611, Boston, MA, USA

[2]Cardiovascular Medicine, Whitaker Cardiovascular Institute, Boston University School of Medicine, 715 Albany Street, E-7 C.H.U., Boston, MA, USA

[3]Department of General Surgery, Boston University School of Medicine, Boston, MA, USA

[4]Department of Medicine, Section of Endocrinology, Diabetes and Nutrition, Boston University School of Medicine, Boston, MA, USA.

Corresponding author:
Kenneth Walsh, Ph.D.
Boston University School of Medicine
715 Albany Street, W-611
Boston, MA, 02118 USA
Phone: 617-414-2390
Fax: 617-414-2391
E-mail: kxwalsh@bu.edu

**Clinical parameter**	All subjects	Non-diabetics	Diabetics
Age (years)	40 ± 2	36 ± 2	47 ± 3
Female sex (%)	73	85	53
BMI (kg/m^2)	45 ± 1	43 ± 2	48 ± 2
Weight (kg)	128 ± 4	122 ± 4	135 ± 8
Waist circumference (cm)	128 ± 3	123 ± 3	134 ± 5
Waist to hip ratio	0.95 ± 0.02	0.92 ± 0.02	0.98 ± 0.03
Fasting glucose (mg/dl)	120 ± 12	90 ± 2	162 ± 23
Plasma Insulin (mIU/ml)	16 ± 2	16 ± 3	12 ± 3
HbA1C (%)	6.1 ± 0.29	5.5 ± 0.1	6.8 ± 0.6
Triglycerides (mg/dl)	122 ± 13	99 ± 7	160 ± 29
Hypercholesterolemia (%)	20	4	47
Total cholesterol (mg/dl)	173 ± 6	175 ± 7	171 ± 10
LDL-cholesterol (mg/dl)	106 ± 4	110 ± 6	99 ± 5
hsCRP (mg/dl)	10.14 ± 1.3	8.76 ± 1.59	12 ± 2
Systolic Blood Pressure (mm Hg)	127 ± 2	125 ± 1	132 ± 4
Diastolic Blood Pressure (mm Hg)	75 ± 1	72 ± 2	76 ± 2
Diabetes (%)	39	0	100
Coronary Heart Disease (%)	4.5	0	12
Hypertension (%)	50	33	76
Hypoglycemic use (%)	29.5	4	76
Lipid lowering use (%)	16	0	41
Anti-inflammatory use (%)	20.5	22	18

Table S1. Study population characteristics. Data expressed as Mean ± SEM

Correlation	Diabetics		Non-diabetics	
	r	p	r	p
ROR2/VANGL2	0.68	< 0.01	0.68	< 0.01
ROR2/FZD7	0.76	< 0.001	0.54	< 0.01
ROR2/ PRICKLE1	0.64	< 0.01	0.54	< 0.01
ROR2/DSH1	0.62	< 0.01	0.03	n.s.
ROR2/DSH2	0.52	< 0.05	0.01	n.s.
ROR2/DSH3	0.52	< 0.05	0.23	n.s.
ROR2/ANKRD6	0.68	< 0.01	0.23	n.s.
VANGL2/FZD7	0.67	< 0.01	0.53	< 0.01
VANGL2/PRICKLE1	0.77	< 0.001	0.81	< 0.0001
VANGL2/DSH1	0.91	< 0.0001	0.55	< 0.01
VANGL2/DSH2	0.89	< 0.0001	0.55	< 0.01
VANGL2/DSH3	0.86	< 0.0001	0.61	< 0.01
VANGL2/ANKRD6	0.90	< 0.0001	0.66	< 0.001
FZD7/PRICKLE1	0.72	< 0.001	0.22	< 0.05
FZD7/DSH1	0.58	< 0.05	0.002	n.s.
FZD7/DSH2	0.52	< 0.05	0.04	n.s.
FZD7/DSH3	0.51	< 0.05	0.11	n.s.
FZD7/ANKRD6	0.72	< 0.01	0.16	n.s.
WNT5A/VANGL2	0.55	< 0.05	0.03	n.s.
WNT5A/PRICKLE1	0.50	< 0.05	-0.03	n.s.
WNT5A/DSH1	0.64	< 0.01	0.16	n.s.
WNT5A/DSH2	0.60	< 0.05	-0.06	n.s.
WNT5A/DSH3	0.66	< 0.01	0.16	n.s.
WNT5A/ANKRD6	0.53	< 0.05	0.03	n.s.

Table S2. Correlations between the expression of WNT5A/PCP signaling genes in visceral fat of diabetic and non-diabetic individuals. Pearson's correlation coefficients (r) were used to analyze the association between variables. n.s.: non-statistically significant ($p>0.1$)

Correlation	Diabetics		Non-diabetics	
	**r**	**p**	**r**	**p**
WNT5A/IL6	0.80	< 0.001	0.46	< 0.05
DSH1/IL6	0.50	< 0.05	0.09	n.s.
DSH2/IL6	0.49	< 0.05	0.18	n.s.
DSH3IL6	0.57	< 0.05	0.03	n.s.
WNT5A/waist-to-hip ratio	0.59	< 0.05	0.43	0.052
DSH1/waist-to-hip ratio	-0.09	n.s.	-0.33	n.s.
DSH2/waist-to-hip ratio	-0.22	n.s.	-0.36	n.s.
DSH3/waist-to-hip ratio	-0.07	n.s.	0.06	n.s.
WNT5A/hsCRP	0.54	0.056	0.01	n.s.
DSH1/hsCRP	0.56	< 0.05	-0.30	n.s.
DSH2/ hsCRP	0.47	0.10	-0.10	n.s.
DSH3/ hsCRP	0.52	0.07	-0.22	n.s.

Table S3. Correlations between the expression of *WNT5A* and *DSH1,2,3* in visceral fat and *IL6* expression in this depot, waist-to-hip ratio and circulating CRP levels in diabetic and non-diabetic individuals. Pearson's correlation coefficients (r) were used to analyze the association between variables. n.s.: non-statistically significant (p>0.1)

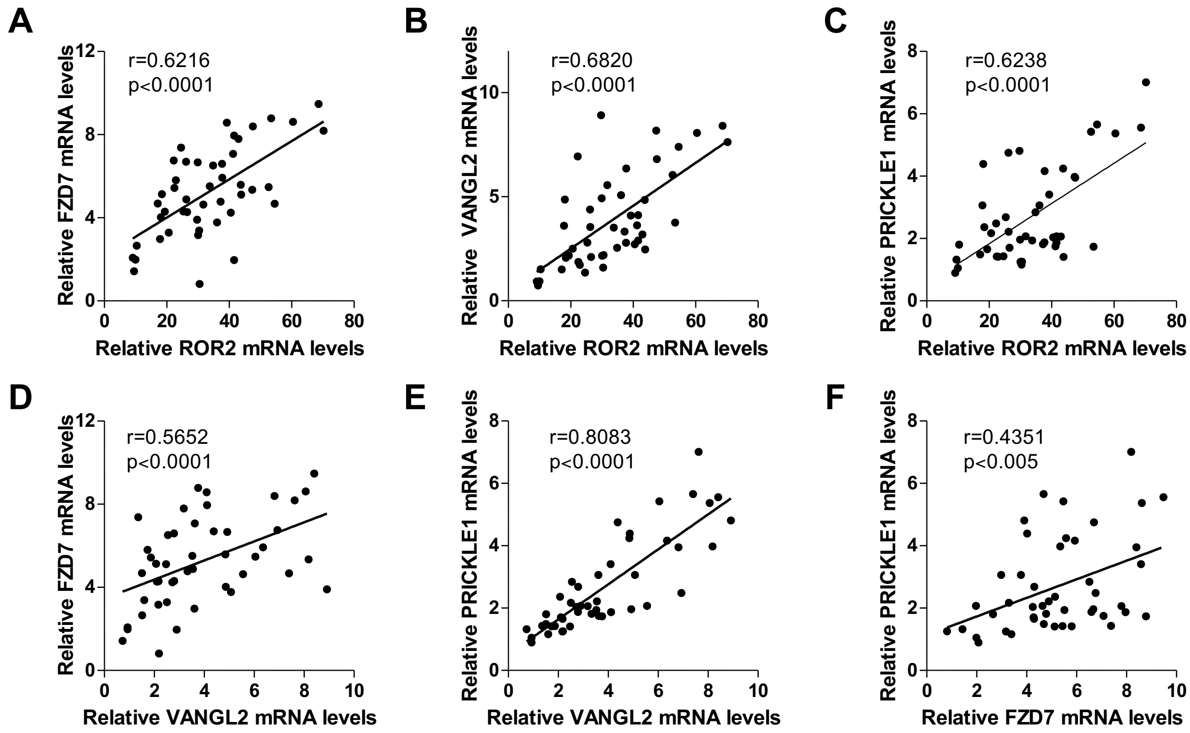

Figure S1. Coordinated upregulation of PCP core receptor components in visceral adipose tissue.

A-F. Pearson's coefficients (r) were used to analyze the correlation between the expression of main PCP receptor components in VAT.

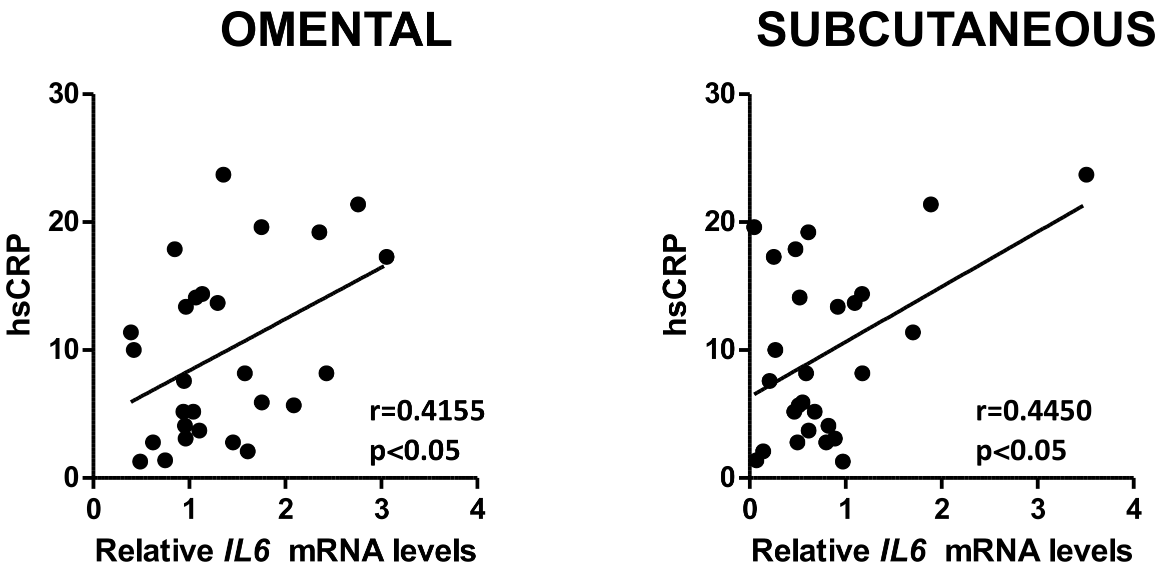

Figure S2. Positive correlation between *IL6* expression and hsCRP in VAT and SAT. Pearson's coefficients (r) were used to analyze the correlation.
